# Strategic elements of residency training in China: transactional leadership, self-efficacy, and employee-orientation culture

**DOI:** 10.1186/s12909-019-1792-7

**Published:** 2019-09-14

**Authors:** Guangwei Deng, Di Zhao, Jonathan Lio, Xinyu Chen, Xiaopeng Ma, Liang Liang, Chenpeng Feng

**Affiliations:** 1 School of Management, Hefei University of Technology, Hefei, 230009 Anhui China; 2 School of Management, University of Science and Technology of China, Hefei, 230026 Anhui China; 3 Department of Medicine, University of Chicago, Chicago, IL 60637 USA; 4 National Health Commission of the People’s Republic of China, Beijing, 100044 China; 5 Division of Life Sciences and Medicine, The First Affiliated Hospital of USTC, University of Science and Technology of China, Hefei, 230001 Anhui China

**Keywords:** Transactional leadership style, Self-efficacy, Employee-oriented organizational culture, Training performance

## Abstract

**Background:**

The standardized training of resident physicians in China is significant and robust. During the training, clinical teachers act as leaders. The training taking place in public hospitals requires a transactional leadership style (TLS), but existing research studies seldom analyze how to promote residents’ performance from this perspective.

**Methods:**

Two hundred and ninety six new residents undertaking standardized training were recruited from five tertiary hospitals in two provinces of China. Hierarchical moderated and mediated regression analyses were used to test the hypotheses. The hypotheses include that TLS is positively related to the training performance; mediating effect of self-efficacy and moderating effect of employee-orientation organizational culture (EOC) are significant.

**Results:**

(1) Two kinds of teachers’ TLS, punishment and reward, have significant positive influence on residents’ performance. (2) Self-efficacy of residents partly mediates the positive relationship. (3) EOC moderates the relationship between the punitive behavior of clinical teachers with TLS and the self-efficacy of the residents.

**Conclusions:**

Empirical evidence has shown the positive relationship between teachers’ TLS and residents’ performance outcomes in China. Teachers can enhance training performance by promoting self-efficacy of residents. This study also advances our understanding of EOC by examining the demonstrated moderating effects of cultural background in the relationship between teachers’ TLS and the self-efficacy of residents.

## Background

Residency training is essential for medical students to become competent doctors by mastering the necessary skills and concepts [1, 2]. There was no standardized residency training system in China for a long period, thus seriously limiting the improvement of the overall quality of the medical teams. Since the 1980s, China considered constructing a standardized training system to solve problems such as a lack of general practitioners, irrational distribution of physicians, and insufficient competence of physicians [3, 4]. The system was not officially established until 2014. The training has begun to work in China, but most existing research has focused on the standard training process rather than on physicians’ capacities to improve professional quality and performance [5]. China’s large population exacerbates the problem of insufficient supply of medical resources due to the increasing aging population, the full liberalization of the two-child policy, and a growing awareness of public health [6]. As a result, China’s health system and medical culture are very different from those in the West [7]. These different professional demands mean that the practical work experience of residents in China is very different from that in the West.

There are few researches from the perspective of organizational behavior on the interactions between trainers (clinical teachers) and trainees (residents) during residency training. As a key disseminator of knowledge, skills, and values in medical practice, clinical teachers effectively determine the training quality [8]. They are also needed to act as administrative leaders in clinical departments, a capacity in which behavioral style is important for residency training [9]. The teachers’ different leadership styles can have different effects on the performance of residents [10]. Clinical teachers in public hospitals prefer transactional leadership style (TLS) in their thinking and behavior patterns [11]. TLS refers to a model wherein leaders reward employees for their performance or take corrective measures for their under-performance, based on the core concept of contingency reward or contingency punishment [12].

Because of the planned system and managerial mechanism, which had worked for long-term periods in the past, existing state-owned institutions, including public hospitals, are still subject to orders and fulfill tasks for compensation and promotion provided by the leaders [13]. Having reconsidered China’s behavioral theory and the rise of localization [14], the academic community recognizes that TLS is not out of date [15], although, in the process of reforming governance mechanism, public organizations strongly advocate transformational leadership. Last, a large number of empirical studies have deemed that TLS is compatible with China’s situation [15] and can yield positive results [16].

**Fig. 1:**
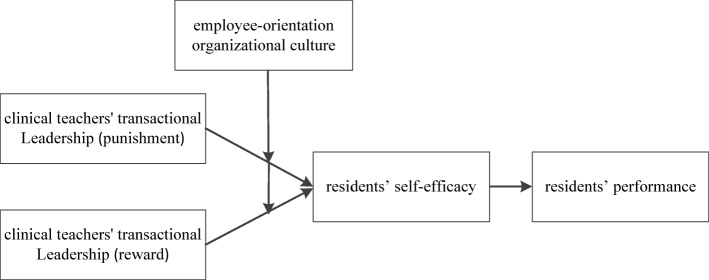
Holistic hypothetical mode

This study was aimed at investigating the relationship between TLS and residents’ performance to further explore the underlying mechanism (See Fig. 1). Specifically, we intend to examine the role of self-efficacy as a link between teachers’ TLS and residents’ performance. Self-efficacy is defined as an individual’s confidence or belief in their ability to achieve behavioral goals in a specific field [17]. As noted by Nielsen and Munir [18], this quality is vital to residents’ performance, and it is considered a significant mediator in the study of leadership style. We also identify moderating mechanisms owing to external cultural factors such as an employee-oriented organizational culture (EOC). EOC is also used to emphasize that employees are the most important resource for the organizations and a value-adding type of capital [19]. However, a universally accepted theoretical model of interactions between clinical teachers and residents has not yet been put forward [10]. Importantly, combining TLS, cultural orientation, and self-efficacy into the same mode contributes to a better understanding of the “black box” problem in medical education [20].

## Methods

### Hypotheses

#### TLS and residents’ training performance

Clinical teachers’ TLS emphasizes training goals, working standards, health outputs & processes, immediate performance, and rewards or negative reinforcement to influence residents. In accordance with the TLS theory [12, 21], teachers reward residents who accomplish work tasks; otherwise, if the residents’ behavior does not meet clinical safety standards, the teacher will correct the residents’ work problems. These medical malpractices from residents can cause harm to patients. Residents will be informed of the potential legal risks and sanctions for such malpractice by the teacher. As a result, TLS with efficiency-oriented and standards-based attributes has a positive impact on performance.

Clinical teachers with TLS try to establish a formal or informal contract with residents. They arrange tasks for residents, and residents remain compliant with the stated goals and plans. For example, teachers are willing to help some well-performing residents to earn more job opportunities in the future. Training bases are usually located in the best hospital in the area. Staying in these hospitals after training means a higher income, more career opportunities, etc. These rewards enable residents to become more confident in their abilities and more willing to participate in training to be competent enough for more clinical work. When residents slack off, they may be criticized by teachers, facing lower evaluation score and unfavorable career prospects.

Residents with different performance levels face different treatment and professional development opportunities provided by their teachers, affording them a broader variety of experiences with others [22]. It makes them realize the importance of striving for performance and take actions. Therefore, TLS has a profound impact on training quality.

- **Hypothesis 1:** The two methods employed by clinical teachers with TLS, contingent punishment and reward, are positively related to the training performance of residents.

#### The mediating effect

People with high self-efficacy establish higher goals, choose challenging jobs, attribute success to their own abilities, and deliver high-quality performance [23]. Self-efficacy mainly affects the trainee’s process of choice, cognition, motivation, and emotion, and also encourages them to reach their potential [24]. Studies have also shown that some behavioral traits of TLS are associated with increasingly higher self-efficacy and further verify that reward-based practices improve self-efficacy [25].

In the field of health, the research on self-efficacy mainly focuses on the effect of improving a certain skill of a trainee [26]. Self-efficacy theory is based on the dynamic interactions between individuals, environments, and behaviors. Unfortunately, self-efficacy has not been clearly identified in studies on the interaction mechanisms between medical students or residents and the environment. Nor has a convincing theoretical model been formed. Nevertheless, we believe that the mediating effect based on the teacher-resident interaction in the residents’ training is extremely important.

The fundamental logic is that when residents achieve training goals, teachers will give them appropriate rewards, thereby establishing an association between hard work and expected accomplishment. Hence, residents will link their personal interests with the benefits from teachers, which will motivate them to work hard to accomplish established goals and become successful. Put another way, external incentives are used to motivate residents to exert their self-efficacy to deliver a high performance and realize the goal of the team. If a resident receives reward or punishment from the teacher, their self-efficacy will either increase or decrease accordingly, which will lead to an increase or decrease in learning performance [27].

- **Hypothesis 2:** Self-efficacy of residents mediates the relationship between the TLS of clinical teachers and the training performance of residents.

#### Moderating effect of EOC

EOC is a critical environmental element for hospitals. Relevant EOC literatures [28] have shown the significance of implementing performance-based incentive measures and career development systems based on employee needs [29].

To promote effective training results, certain environmental conditions, such as favorable clinical culture orientation, must be satisfied [30]. George [31] believes that internal validity is a prerequisite for external validity. If clinical teachers want residents to provide better service when interacting with patients, hospitals must first provide better service to residents. The EOC perspective is that the hospitals should trust and empower their residents as much as possible, allowing them to participate extensively in clinical activities [28]. EOC is important as a supportive factor in residency training. Because positive environmental factors can enhance the role of teachers’ leadership behavior and residents’ self-efficacy, it promotes training performance. This reveals the fact that EOC acts as a potential moderator.

During the training process, the leaders, especially teachers in public hospitals who have frequent contact with residents, should adhere to the management concept of respect for employees. With residences at the center, it helps them gain vicarious experience by observing the practices of regular employees, achieve a sense of self-existence, and participate more actively in their work. A sense of belonging is weaker in residents who are external employees that require more emotional support from hospital organizations and members. Hospitals with strong EOC treat residents equally, allowing them to frequently exchange views with clinical teachers. This relationship benefits their professional capability and emotional state, supporting them in meeting the prerequisites for high-achieving training performance. Based on these actions, hospitals’ EOCs not only promote the realization of training objectives, but also play a positive role in clinical teachers’ TLS while downplaying the potential risks of transactional leadership behavior.

- **Hypothesis 3:** The moderating effect of the EOC on the clinical teachers with TLS and self-efficacy of resident is significant.

### Sample selection and variable measurement

#### Samples and procedures

In 2017, we conducted a survey on residents in the first batch of standardized residency training bases in China. These bases are located in five tertiary hospitals in central and eastern China. These institutions were chosen as the only hospitals qualified as training bases certified by the National Health and Family Planning Commission. We contacted the hospital via phone or social network to obtain permission to start the investigation. We communicated with the staff of the Office of Resident Management or other informal organizations of residents (such as alumni associations) and asked them to provide detailed information about the residents. In order to avoid crossing, we reminded and advised them to fill in TLS situation of the teacher in their own professional department.

We presented a total of 400 paper surveys. Residents were asked to answer anonymously, and the surveys were collected on the spot. All the residents were well-informed about the contents and the aim of the questionnaire, which encouraged them to complete it independently. Residents from various disciplines including pediatrics, emergency medicine, internal medicine, and others took part in the survey. A total of 296 questionnaires were collected, excluding 49 that were deemed invalid due to missing information or inconsistent statements. And the remaining 247 were used for data analysis, yielding an effective participation rate of 61.7%. Considering that residents differ in their educational backgrounds, age, and other factors, we control these factors as demographic variables in the questionnaire.

#### Variable measurement

The questionnaire consists of four general international scales to measure TLS, self-efficacy, EOC and training performance of residents. All use the 5-point Likert-Type Scale, which is divided into 1 (very disagree) to 5 (very agree).

**TLS** A four-item contingent reward behavior scale [21, 32] was used to assess the degree to which a leader provides positive feedback. Contingent punishment was assessed with three items from Podsakoff et al.’s [21] contingent punishment scale.

**Self-Efficacy** The New General self-efficacy Scale of the 8 items of Self-efficacy is used, it demonstrated high reliability, predicted specific self-efficacy for a variety of tasks in various contexts, and moderated the influence of previous performance on subsequent residual sum of squares (SSE) formation [33].

**EOC** The Tsui scale has been used several times to measure organizational culture in China [34]. In the survey of Chinese hospital organizational culture, stability of reliability and validity was shown [35]. The EOC dimension includes six items such as concerning for the individual development of employees and caring about opinions from employees.

**The performance of residents** In order to help assess resident performance during training, the Residency Affair Committee of the Pediatric Residency Program of the University of Padua (Italy) administered a resident assessment questionnaire (ReAQ), and it consists of 20 items that assess the six core competencies identified by the Accreditation Council of Graduate Medical Education [36].

## Results

### Reliability and validity

All hypotheses were tested by hierarchical regression analyses via SPSS 19.0. Confirmatory factor analysis was used to assess the reliability and validity. See Table 1. Construct reliability was assessed by Cronbach’s Alpha and composite reliability. Cronbach’s α ranges from 0.76 to 0.94, composite reliability ranges from 0.87 to 0.95, which are both above the 0.70 recommended levels, indicating satisfactory construct reliability.

**Table 1:** Loadings, Cronbach’s α, Composite Reliability and AVE

Variables	Loading	Cronbach’s *α*	Composite Reliability	AVE
Self-efficacy	0.61–0.78	0.86	0.89	0.51
TLS (contingent reward)	0.85–0.89	0.85	0.91	0.77
TLS (contingent punishment)	0.78–0.86	0.76	0.87	0.69
Organizational culture	0.66–0.85	0.94	0.95	0.58
Performance	0.61–0.81	0.92	0.94	0.50

Convergent validity was assessed by the factor loadings and the average variance extracted (AVE). All item loadings are higher than the 0.60 criterion at a significance level of 0.001, and the AVE scores were higher than or equal to the benchmark value of 0.50, indicating acceptable convergent validity.

Discriminant validity was tested by comparing the relationship between the correlations among constructs and square root of the AVE scores. Table 2 (indicates that the square root of the AVE scores for each construct is greater than the correlations among the constructs, thus confirming the discriminant validity.

**Table 2:** Means, standard deviations, correlations

Variable	AV	SD	1	2	3	4	5	6	7	8	9
Gender											
Age			0.02								
Education			−0.06	0.18**							
Hospital level			0.06	−0.01	− 0.27**						
Self-efficacy	3.65	0.49	0.12*	0.05	0.00	0.04	(0.71)				
TLS (contingent punishment)	3.61	0.61	0.19**	0.00	0.12	0.09	0.14	(0.88)			
TLS (contingent reward)	3.58	0.66	0.03	0.04	−0.01	−0.04	0.31**	−0.05	(0.83)		
EOC	3.51	0.59	−0.14*	0.07	−0.12	0.03	0.39**	0.07	0.49**	(0.76)	
Performance	3.61	0.47	−0.00	0.09	0.02	0.07	0.51**	0.24**	0.45**	0.63**	(0.71)

Notes: *n* = 247. The diagonal elements are the square root of AVE. **p < 0.01; *p < 0.05

### Hypotheses tests

#### Main effect

The main effect is to test the influence of teachers’ TLS on residents’ training performance. According to the linear regression analysis step, the control variables are first entered, and then independent variables. The model 4 of Table 3 shows that: two types of TLSs: contingent reward (β = 0.47, *p* < 0.001) and contingent punishment (β = 0.27, *p* < 0.001) all have significant positive impacts on internship performance, supporting Hypothesis 1.

**Table 3:** Results of regression analysis for mediating effects

	Self-efficacy		Performance		
	Model 1	Model 2	Model 3	Model 4	Model 5
Gender	0.12	0.09	−0.01	−0.07	− 0.11*
Age	0.04	0.03	0.08	0.07	0.06
Education	0.00	−0.01	0.02	−0.01	− 0.01
Hospital level	0.03	0.03	0.07	0.06	0.05
TLS (contingent reward)		0.31***		0.47***	0.35***
TLS (contingent punishment)		0.14***		0.27***	0.22***
Self-efficacy					0.38***
*R* ^*2*^			0.01	0.29	0.41
*△R* ^*2*^			0.01	0.27	0.13
*F*			0.75	15.93***	23.78***
*△F*			0.75	45.75	50.99

Note: *n* = 247. ***p < 0.001; *p < 0.05

#### Mediating effect

To analyze the mediating effect of self-efficacy in the relationship between TLS and training performance, control variables are the first to put into the regression equation, then the mediation variables, finally, both the independent variables and the mediation variables are added. In Table 3, contingent reward was significantly related to self-efficacy (β = 0.31, *p* < 0.001), the same as contingent punishment (β = 0.14, *p* < 0.001). The effects of contingent reward (β = 0.35, *p* < 0.001) and contingent punishment (β = 0.22, *p* < 0.001) on internship performance were smaller but still significant when incorporating self-efficacy. Hence, we deduce that self-efficacy plays a partially mediating role, supporting Hypothesis 3.

#### Moderating effect

For the moderating effect of EOC on teachers’ TLS and residents’ self-efficacy: We first set the self-efficacy as the dependent variable, then we add control variables, independent variables, moderating variables, the interactions of independent variables and moderating variables into the regression equation. Both independent and moderating variables are centralized to avoid multicollinearity.

**Fig. 2:**
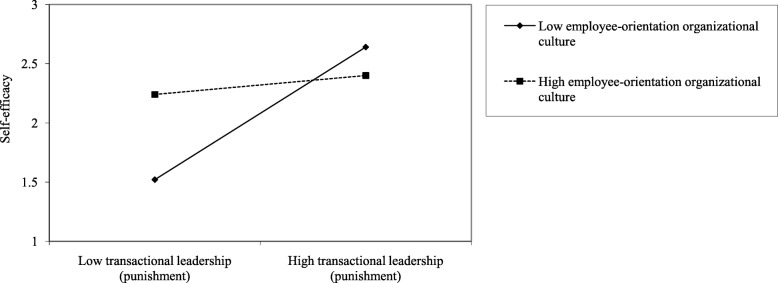
Interaction between the punishment behavior of clinical-teachers with TLS and self-efficacy of residents

As shown in Model 9 of Table 4, EOC plays a significant role in the punishment behavior of clinical teachers with TLS and self-efficacy of residents (β = − 0.24, *p* < 0.001). It indicates that, in the context of low EOC level, the promotion of the punishment behavior of clinical teachers with TLS to self-efficacy of residents increase, that is, hypothesis H3 is established. The moderating effect of the EOC on the rewarded behavior of clinical teachers with TLS and self-efficacy of residents is not significant (β = 0.00, p>0.1). This indicates that, regardless of the high or low EOC level, the rewarded behavior of teachers with TLS does not change the self-efficacy of residents. See Fig. 2.

**Table 4:** Results of regression analysis for moderating effects

	Self-efficacy			
	Model 6	Model 7	Model 8	Model 9
Gender	0.12	0.09	0.15*	0.17**
Age	0.04	0.03	0.01	0.02
Education	0.01	−0.01	0.05	0.04
Hospital level	0.03	0.03	0.03	0.03
TLS (contingent reward)		0.31***	0.08	0.04
TLS (contingent punishment)		0.14***	0.34***	0.32***
EOC			0.14*	0.12
TLS (contingent reward)-*EOC				0.00
TLS (punishment)*-EOC				−0.24***
*R* ^*2*^	0.02	0.13	0.21	0.26
*△R* ^*2*^	0.02	0.11	0.08	0.06
*F*	1.09	5.76***	8.86***	9.28***
*△F*	1.09	14.85	24.09	8.75

Notes: *n* = 247. ***p < 0.001; **p<0.01; *p < 0.05

In short, the main conclusions are as follows: (1) The two methods employed by clinical teachers with TLS, punishment and reward, significantly promote the training effect of residents; (2) Self-efficacy of residents plays a partly mediator role between the TLS of teachers and the training performance of residents; (3) EOC negatively moderates the relationship between punishment behavior of teachers with TLS and self-efficacy of residents, but has no moderating effect on the relationship of reward behavior of teachers with TLS and self-efficacy of residents.

## Discussion

First, the findings of this study are consistent with the prior research standing that TLS contributes to performance [37]. Compared with Haoka’s research [38], which rewards residents, our study explores the role of negative reinforcement in achieving performance. In particular, the punishment is not corporal in nature, nor is it disrespectful or insulting to residents; rather, it is a way to educate and warn residents to reduce the possibility or tendency toward malpractice. The punishment used here is a kind of corrective action, an acceptable educational measure taken by clinical teachers. The purpose of the training is to enable trainees to clearly understand their behavioral boundaries, strictly enforce the standards, properly handle the relationship between clinical rights and responsibilities, and to enhance the awareness of rules. According to Bandura’s social learning theory, when one resident is criticized for misconduct, the critical behavior will strengthen their standardized behavior. Meanwhile others are also indirectly learning through the action of criticism.

Second, this study explores a mediation model whereby clinical teachers’ TLS impacts training performance via self-efficacy. Unlike in previous studies, self-efficacy is used as an independent variable [26, 39]. In our research, based on the theoretical model of individual, environmental, and behavioral dynamic interactions, the mediating role of self-efficacy is constructed to make the model closer to the real situation. The training performance of residents depends largely on the intrinsic impetus to achieve their own self-efficacy in learning, and TLS enhances residents’ enthusiasm to complete training, prompting them to achieve high-quality performance in a short period by clarifying learning objectives and reward methods. The research has also validated self-efficacy as an effective mediator.

Third, we examined the moderating effect of employee-oriented hospital culture. EOC is negatively related to the punitive behavior of clinical teachers with TLS and self-efficacy of residents. Our results show that hospitals with weak EOC are more likely to rely on the method of punitive behavior. Contrarily, in hospitals with strong EOC, residents are respected and seldom punished. In order to avoid punishment, residents will take the initiative to complete the task of training in accordance with the requirements of teachers. When the tasks are finished, their self-efficacy will be increased. In addition, survey results showed that residents strongly look forward to building an employee-oriented hospital. Like other countries [40, 41] that have carried out residents’ training, China is also facing the challenge of residents’ burnout. Because of their long working hours, heavy workload, and highly intensive work, residents are routinely exhausted [42, 43]. It is necessary to build an effective organizational culture to reduce this burnout [44]. Hospitals should respect, help, and actively communicate with residents. EOC should be established to reduce the occurrence of training burnout and improve residents’ performance.

## Conclusions

In short, the two methods employed by clinical teachers with TLS significantly promote effective training in residents. However, we do not advocate punishment, because it may exacerbate work stress and job burnout among residents. Self-efficacy of residents plays a mediating role between the TLS of teachers and the training performance of residents. EOC negatively moderates the relationship between punitive behavior of teachers with TLS and self-efficacy of residents but has no moderating effect on the relationship of rewarding behavior of teachers with TLS and self-efficacy of residents.

This paper has several limitations. The first is its utilization of horizontal reporting data. The effects of some variables on others require time to be revealed. Vertical (time-series) data will yield more convincing conclusions. However, due to practical considerations, we used a cross-sectional design. Vertical data can be used in future studies to test the model proposed here. A second limitation owes to the cultural differences among participants in our samples. Future research can expand its sampling method to observe the functional mechanisms of teachers’ leadership styles and performance of residents representing different economic levels, within different policy environments, and with both Chinese and Western cultural backgrounds. A third limitation is the result that EOC does not significantly moderate the relationship between the rewarding behavior of teachers’ TLS and the self-efficacy of residents. Thus, it would be interesting for future research to explore the influence of other potential moderating and mediating variables.

## Data Availability

The datasets used and/or analysed during the current study are available from the corresponding author on reasonable request.

## Abbreviations

AVE: Average variance extracted
EOC: Employee-oriented organizational culture
ReAQ: Resident assessment questionnaire
SSE: Residual sum of squares
TLS: Transactional leadership style
